# Protistan Predators Outshine Fungi in Forest Soil Activity

**DOI:** 10.1111/jeu.70072

**Published:** 2026-03-31

**Authors:** Longfei Kang, Kenneth Dumack

**Affiliations:** ^1^ Terrestrial Ecology, Institute of Zoology, University of Cologne Köln Germany; ^2^ Shaanxi Key Laboratory of Ecological Restoration in Shaanbei Mining Area, College of Advanced Agricultural Sciences, Yulin University Yulin China; ^3^ University of Koblenz Aquatic Ecosystem Analyses Institute for Integrated Natural Sciences Koblenz Germany

**Keywords:** Arcellinida, forest soil ecosystems, metatranscriptomics, microbial communities, microbial ecology

## Abstract

Despite extensive research on fungal communities in forest soils, our understanding of the whole eukaryotic diversity and distribution remains limited. Moreover, traditional amplicon sequencing methods often introduce severe PCR and primer biases, further hindering accurate assessment of the microbial community composition in forest soils. To address these challenges, this study used a public metatranscriptomic data set to analyze 51 forest soil samples comprising four countries (Canada, France, Spain, and Sweden). Our results reveal that *Arcellinida*, a eukaryotic order of shell‐bearing amoebae, represent the most abundant eukaryotic taxon in forest soils, with an average relative abundance of 12.6%. This finding challenges the conventional view that fungi dominate eukaryotic diversity in these ecosystems. Furthermore, our study demonstrates that *Arcellinida* (*R* ^2^ = 0.066, *p* = 0.006) and soil pH (*R* ^2^ = 0.126, *p* < 0.001) are key biological and environmental drivers, respectively, shaping the composition of eukaryotic communities in forest soils, suggesting distinct impact on the microbial community through predation. These findings offer novel insights into the ecological significance of microbial eukaryotes in forest ecosystems and provide a new framework for investigating the predatory dynamics centered on *Arcellinida* in forest soil microbial networks.

## 1 Introduction

Forests play a crucial role in terrestrial ecosystems, contributing significantly to global carbon sequestration, biodiversity conservation, and climate regulation (Khadakkar 2022; Leal Filho et al. 2023; Liu et al. 2025). They are often referred to as the lungs of the Earth, providing essential ecosystem services such as water purification, soil stabilization, and habitat provision for a vast array of species. Forest soils, as a complex and dynamic environment that supports a diverse array of life forms, are considered biodiversity hotspots, hosting a wide range of plant, animal, and microbial species (Meena et al. 2023). Microorganisms (e.g., bacteria, fungi, protists and archaea) play essential roles in nutrient cycling, organic matter decomposition, and soil structure formation, thereby influencing forest soil multifunctionality and productivity (Bastida et al. 2021; Fierer and Jackson 2006). In this century, fungi have become one of the most comprehensively and thoroughly investigated eukaryotic communities in forest ecosystems due to their important ecological functions and roles, including decomposing organic matter, forming mutualistic relationships with terrestrial plants, and protecting their host plants against pathogens (Auer et al. 2024; Bahram et al. 2016; Mundra et al. 2021; Wu et al. 2019). However, large‐scale investigations into the diversity and ecological functions of other eukaryotic groups remain limited, and their ecological roles are still poorly understood to date.

Protists are polyphyletic single‐celled eukaryotes and key components of soil food webs, acting as predators of bacteria and fungi (Bonkowski 2004; Clarholm 1985; Estermann et al. 2023; Geisen et al. 2015). Obviously, they play important roles in controlling microbial community structure and function, which in turn affects soil fertility and ecosystem stability in forest soils (Geisen et al. 2018; Oliverio et al. 2020). Arcellinida, commonly known as testate amoebae, are a group of protists belonging to the phylum Amoebozoa. They are widely distributed in terrestrial ecosystems and characterized by their unique, often rigid shells that provide both protection and a means for identification (Dumack et al. 2024). This distinctive feature sets them apart from other protists and makes them valuable subjects for ecological and evolutionary studies. Moreover, Arcellinida act as top predators in microbial food webs, regulating bacterial and fungal populations (García‐Bodelón et al. 2025), and it is hypothesized that their shell functions as a microbial weapon enabling them to rupture large prey like fungal hyphae and nematodes (Dumack et al. 2024; Estermann et al. 2023). Numerous studies have shown that these organisms are primarily identified and enumerated morphologically and are routinely used to provide insights into both the stability and the general ecological health of specialized environments (e.g., soils, sediments, bogs and lakes) (García‐Bodelón et al. 2025; González‐Miguéns et al. 2024; Patterson and Kumar 2002; Useros et al. 2024). They are routinely used for specific applications such as monitoring perturbations in peatland restoration management (Carballeira and Pontevedra‐Pombal 2021; Marcisz et al. 2016) and to evaluate the impact of anthropogenic disturbance on soil properties (Heger et al. 2013; Wanner and Dunger 2001). There is thus an extensive scientific literature on the sensitivity of these organisms to different environmental perturbations (Freitas et al. 2022). Arcellinida also serve as sensitive bioindicators of environmental changes, reflecting disturbances caused by pollution, climate variations, and land management practices (Su et al. 2024). Additionally, these organisms contribute to organic matter decomposition and participate in the carbon and nitrogen cycles (Dufour et al. 2024). Their feeding activities break down complex organic compounds, releasing nutrients back into the soil, thus promoting nutrient availability for plants and other organisms (Heger et al. 2012). Other literature emphasized that Arcellinida also can function as a model group for studying microbial biogeography (Singer et al. 2019; Smith and Wilkinson 2007). The ecological roles of Arcellinida highlight their importance in maintaining the stability and function of terrestrial ecosystems. Therefore, understanding the distribution, community dynamics, and ecological interactions of Arcellinida is essential for developing effective conservation and management strategies.

The traditional classification of Arcellinida relies heavily on morphological characteristics, particularly the size, shape, and composition of their shells. These shells can be composed of self‐secreted elements (e.g., calcareous, siliceous, or chitinoid) or recycled organic and mineral particles (Dufour et al. 2024; Gomaa et al. 2017; González‐Miguéns et al. 2022). Recently, molecular studies have significantly influenced the classification of Arcellinida. Molecular approaches, such as SSU rRNA gene sequencing, have revealed much greater biodiversity than previously estimated through morphological methods (Mauricio‐Sánchez et al. 2025). For example, studies on genera like *Nebela*, *Difflugia*, and *Hyalosphenia* have identified cryptic lineages that are genetically distinct but morphologically indistinguishable (Dufour et al. 2024). These findings challenge the traditional reliance on shells as a primary taxonomic character and highlight the importance of molecular tools in resolving the phylogenetic relationships within Arcellinida (Gomaa et al. 2017; Kosakyan et al. 2016). While morphological characteristics remain important for the initial identification of Arcellinida, molecular biology has become essential for resolving their phylogenetic relationships and uncovering hidden diversity. Future taxonomic studies will likely continue to integrate morphological and molecular approaches to better understand the complexity of Arcellinida.

In recent years, metabarcoding has significantly expanded our understanding of microbial eukaryotic diversity (Cristescu 2014; Useros et al. 2024), such as the transformative impact of metabarcoding approaches targeting the small subunit ribosomal RNA (18S in eukaryotes) on understanding eukaryotic diversity (Santoferrara et al. 2020). However, these methods exhibit significant biases and limitations when applied to Arcellinida (Berney et al. 2004; Lara et al. 2022). Specifically, the 18S rRNA sequences of Arcellinida exhibit high evolutionary heterogeneity, often containing lineage‐specific structural elements and introns, which can extend the amplicon length beyond the capacity of conventional sequencing platforms (Gomaa et al. 2017; Lara et al. 2008). Additionally, sequencing biases may arise from primer mismatches with specific Arcellinida sequences and the amplicon length exceeding the preferred range of sequencing platforms (Choi and Park 2020; Xie et al. 2011). This technical limitation directly results in a significant underrepresentation of certain subgroups (e.g., Glutinoconcha) in the EukBank database, while the suborder Phryganellina, which contains fewer described species, seems disproportionately represented (González‐Miguéns et al. 2022). Therefore, although metabarcoding remains an effective tool for assessing microbial eukaryotic diversity, fully revealing the true environmental diversity of taxa with complex rDNA structures, such as Arcellinida, requires the integration of alternative sequencing technologies (Berney et al. 2015; Geisen et al. 2015; Lara et al. 2022).

In this study, we used a metatranscriptomic approach to reveal the community composition and diversity of prokaryotic and eukaryotic communities in forest ecosystems on a wide scale (Canada, France, Spain and Sweden). Moreover, the driving mechanisms for diversity of prokaryotic and eukaryotic communities were identified. Our results revealed high relative abundance and diversity of Arcellinida, in which communities differed strongly between sites. This study highlighted the importance of Arcellinida in forest ecosystems for the first time.

## 2 Materials and Methods

### 2.1 Data Access and Zone Description

In this study, we used a publicly available dataset provided by Auer et al. (2024). Briefly, four coniferous forest zones were selected, and 51 soil samples were collected from Montmorency, Canada (Canada_Mrcy, *n* = 9), Champenoux, France (France_Chpx, *n* = 18), Aspurz, Spain (Spain_Aspz, *n* = 12), and Lamborn, Sweden (Sweden_Lbrn, *n* = 12). The forest types in Canada_Mrcy, France_Chpx, Spain_Aspz, and Sweden_Lbrn are boreal, temperate, sub‐Mediterranean, and boreal, respectively. We used these metatranscriptomic data (RNA) to evaluate the prokaryotic and eukaryotic communities in forest ecosystems. A detailed geographical and climatic description of these four coniferous forest zones is provided in Table S1.

In the Canadian Montmorency zone, the forest is dominated by balsam fir ( *Abies balsamea* ), and the altitude ranges between 600 and 1000 m. The soil is classified as Humo‐Ferric Podzols or Orthic (Auer et al. 2024). In the French Champenoux zone, the altitude is 254 m, and the dominant tree species is silver fir ( *Abies alba* ), with ground vegetation consisting of *Fraxinus excelsior* seedlings. The Spanish Aspurz zone is a mixed forest with a Mediterranean climate. The forest is dominated by Scots pine ( *Pinus sylvestris* ), and the altitude is 615 m. In the Swedish Lamborn zone, the predominant tree species is Scots pine ( *Pinus sylvestris* ), and the altitude is approximately 273 m. The mean stem density is 750 ha^−1^, and the soil is characterized as sandy moraine/podzol (Lindahl et al. 2007). For each soil sample, a detailed description of artificial disturbances, management practices, and nutrient status is provided in Table S2.

### 2.2 Bioinformatics Processing

RNA extraction and quality filtering: (i) RNA was extracted as part of a sequential co‐isolation procedure following sample collection. Detailed protocols for RNA extraction and cDNA library preparation are provided in Auer et al. (2024); (ii) The raw metatranscriptomic data quality was evaluated using FastQC v.0.11.9 (Andrews 2010); (iii) TrimGalore v.0.6.7 (Krueger et al. 2021) was employed to identify and remove adapters, trim low‐quality bases (quality score < 30), and discard the last 10 bases at the 3′ end; (iv) Paired‐end reads were assembled into contigs using Mothur v.1.45.3 (Schloss et al. 2009); (v) Contigs were subsequently screened using parameters optimized for the dataset, including a minimum length of 90 bp and a maximum of 2 ambiguous bases (Heck et al. 2023).

After raw data quality control (RDQC), sequences were screened using the BLASTN algorithm (Camacho et al. 2009) against specific databases. Eukaryotic and prokaryotic taxa were identified using the PR2 v5.0.0 database (Guillou et al. 2013) and the SILVA 138 SSU Ref NR99 database (Pruesse et al. 2007), respectively. The best hit for each sequence was retained based on an e‐value threshold of 1e^−35^ for PR2 and 1e^−30^ for SILVA, along with a bit‐score threshold of 140 and an identity threshold above 92% (Heck et al. 2023). Sequences from Archaeplastida (plants) and their chloroplasts were excluded to focus on the forest soil microbial community. Taxon counts were aggregated at the genus level to define OTUs for downstream analyses. Data filtering and analysis were performed in R v.4.0.6 (R Core Team 2021) using the packages tidyr v.1.1.3 (Wickham and Girlich 2022), dplyr v.1.0.7 (Wickham et al. 2022), ape v.5.6‐2 (Paradis et al. 2004), lattice v.0.20‐41 (Sarkar 2008), and vegan v.2.5‐7 (Dixon 2003). Visualization was performed using ggplot2 v.3.3.4 (Wickham 2016).

### 2.3 Visualization and Statistical Analyses

To visualize the variations in prokaryotic and eukaryotic community composition among different forest zones, stacked bar plots of the top 20 most abundant communities (at the order and genus levels) were created using the vegan v.2.5‐7 (Dixon 2003), car v.3.1‐2 (Fox and Weisberg 2018), ggplot2 v.3.3.4 (Wickham 2016), and reshape2 v.1.4.4 (Wickham 2020) packages in R software v.3.8.5. The relative read numbers of forest soil microbial kingdoms were analyzed using Microsoft Office Excel (v.2010) and the Statistical Package for the Social Sciences (SPSS, v.22.0) (Table 1 and Tables S1–S3). To further elucidate the influences of environmental factors and microbial taxa (top 10 most abundant communities at the order level) on the community structure of prokaryotes and eukaryotes, non‐metric multidimensional scaling (NMDS) plots were constructed using the envfit, vegdist, and metaMDS functions from the vegan package (Table S3). These plots were generated based on the Bray‐Curtis distance method (Moebius‐Clune et al. 2013).

**TABLE 1:** Relative read numbers of microbial kingdoms in a forest ecosystem.

Domain	Groups	Canada_Mrcy	Sweden_Lbrn	France_Chpx	Spain_Aspz	Average
		(Percentage, %)				
Prokaryotes	Bacterial	70.5 ± 0.47 a	70.4 ± 0.20 a	70.4 ± 0.36 a	70.4 ± 0.28 a	70.4
	Archaea	0.17 ± 0.02 d	0.29 ± 0.01 d	0.26 ± 0.05 d	0.27 ± 0.10 d	0.25
Eukaryotes	Protists	18.9 ± 1.35 b	22.3 ± 2.14 b	19.6 ± 1.29 b	19.4 ± 1.06 b	20.1
	Fungi	10.4 ± 2.09 c	6.99 ± 1.37 c	9.53 ± 2.16 c	9.61 ± 1.90 c	9.13
	Animal (Metazoa)	0.036 ± 0.024 e	0.036 ± 0.021 e	0.210 ± 0.114 d	0.284 ± 0.087 d	0.14
Prokaryotes/Eukaryotes		70.7/29.3	70.7/29.3	70.7/29.3	70.7/29.3	/

*Note:* Distributions (mean ± SE) of bacteria, archaea, protists, fungi and metazoa at the Canada_Mrcy (*n* = 9), Sweden_Lbrn (*n* = 12), France_Chpx (*n* = 16) and Spain_Aspz (*n* = 12) sites.

### 2.4 Language Editing

The authors used the generative AI tool ChatGPT (OpenAI) solely for superficial language editing (grammar and clarity) in parts of the manuscript. It did not contribute to the generation of scientific content, data interpretation, or conclusions, nor to figure preparation or image manipulation. The authors retain full responsibility for all aspects of the work.

## 3 Results

### 3.1 Sequence Reads Distribution

The taxonomic richness of microbial organisms associated with forest soil was found to be high. Specifically, a total of 11,026,251 SSU rRNA contiguous sequence reads were identified. These sequence reads were assigned to 1527 distinct orders (Table S4) and 4083 distinct species. A consistent distribution pattern of sequence reads was observed across different sampling sites. Despite the differences between various sampling sites, eukaryotes (29.3% of all reads and 31.8% of order richness) were predominantly represented by protists, which accounted for 89.9% of eukaryotic orders and contributed 64.4% of the eukaryotic sequence reads (Table 1 and Table S4). Fungi made up 8.8% of eukaryotic orders and were responsible for 35.5% of the sequence reads, while microscopic Metazoa comprised 1.2% of eukaryotic order richness and contributed 0.13% of the sequence reads. Prokaryotes represented the majority of sequence reads and order richness, with approximately 70.7% and 68.2%, respectively. Among prokaryotes, bacteria accounted for 98.3% of all prokaryotic orders, representing approximately 99.7% of all prokaryotic sequence reads. Archaea represented approximately 1.7% of prokaryotic order richness and contributed approximately 0.36% of all prokaryotic sequence reads.

### 3.2 Microbial Community Composition

**FIGURE 1:**
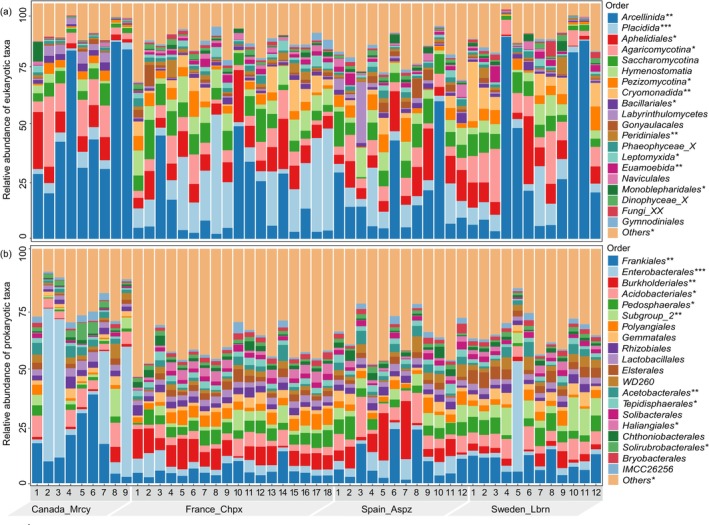
Microbial community composition in different forest soils at the order level. Stacked bar plots display the relative abundance of the 20 most abundant eukaryotic (a) and prokaryotic (b) taxa.

**FIGURE 2:**
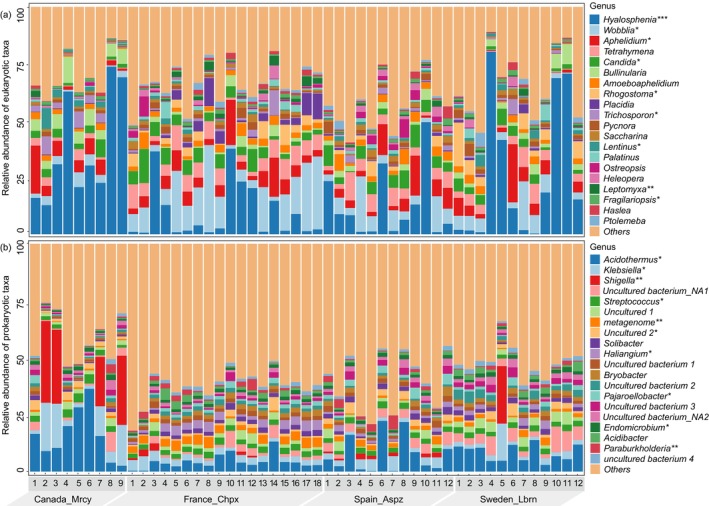
Microbial community composition in different forest soils at the genus level. Stacked bar plots display the relative abundance of the 20 most abundant eukaryotic (a) and prokaryotic (b) taxa. Shigella represents Escherichia–Shigella. Uncultured bacterium NA1 and NA2 remain unidentified at the genus level but have been classified at higher taxonomic levels as *Bacteria_Acidobacteriota_Acidobacteriae_Subgroup_2_uncultured‐bacterium* and *Bacteria_Proteobacteria_Gammaproteobacteria_WD260_uncultured‐bacterium*, respectively. Uncultured 1 and 2 represent *Bacteria_Planctomycetota_Planctomycetes_Gemmatales_Gemmataceae_uncultured* and *Bacteria_Proteobacteria_Alphaproteobacteria_Acetobacterales_Acetobacteraceae_uncultured*, respectively. Uncultured bacterium 1, 2, 3, and 4 represent *Bacteria_Planctomycetota_Phycisphaerae_Tepidisphaerales_WD2101_soil_group_uncultured‐bacterium, Bacteria_Proteobacteria_Alphaproteobacteria_Elsterales_uncultured_uncultured_bacterium, Bacteria_Acidobacteriota_Acidobacteriae_Acidobacteriales_uncultured_uncultured_bacterium*, and *Bacteria_Acidobacteriota_Acidobacteriae_Acidobacteriales_uncultured_uncultured_bacterium*, respectively.

Moreover, at the order level, the composition of eukaryotic community was dominated by *Arcellinida* (Protists_Amoebozoa), making up 25.4% of all eukaryotic rRNA reads, followed by *Placidida* (10.4%, Protists_TSAR), *Aphelidiales* (8.6%, Fungi_Obazoa), *Agaricomycotina* (7.62%, Fungi_Obazoa), *Saccharomycotina* (6.2%, Fungi_Obazoa), *Hymenostomatia* (4.7%, Protists_TSAR), *Pezizomycotina* (3.6%, Fungi_Obazoa) and *Cryomonadida* (3.1%, Protists_TSAR). The prokaryotic community was dominated by *Frankiales* (8.9%, Bacteria_Actinobacteriota), followed by *Enterobacterales* (8.3%, Bacteria_Proteobacteria), *Burkholderiales* (5.2%, Bacteria_Proteobacteria), *Acidobacteriales* (5.1%, Bacteria_ Acidobacteriota), *Pedosphaerales* (4.2%, Bacteria_Verrucomicrobiota), Subgroup_2 (3.8%, Bacteria_Acidobacteriota), *Polyangiales* (3.1%, Bacteria_Myxococcota), and *Gemmatales* (2.5%, Bacteria_Planctomycetota) (Figure 1). At the genus level, *Hyalosphenia* (19.1%, Protists_ Amoebozoa) predominated in all eukaryotic rRNA reads, followed by the second most abundant genus *Wobblia* (8.8%, Protists_TSAR). Other highly abundant groups included *Aphelidium* (5.9%, Fungi_Obazoa), *Tetrahymen*a (4.7%, Protists_TSAR), *Candida* (4.5%, Fungi_Obazoa), and *Bullinularia* (2.8%, Protists_Amoebozoa), while the prokaryotic community was dominated by *Acidothermus* (8.0%, Bacteria_Acidobacteriota), followed by *Klebsiella* (4.2%, Bacteria_ Proteobacteria), *Shigella* (3.26%, Bacteria_Proteobacteria), Uncultured bacterium NA1 (2.9%, Bacteria_Acidobacteriota), *Streptococcus* (2.4%, Bacteria_Firmicutes), and Uncultured_1 (2.2%, Bacteria_Planctomycetota) (Figure 2).

### 3.3 Environmental and Biological Factors Shape Microbial Communities

**FIGURE 3:**
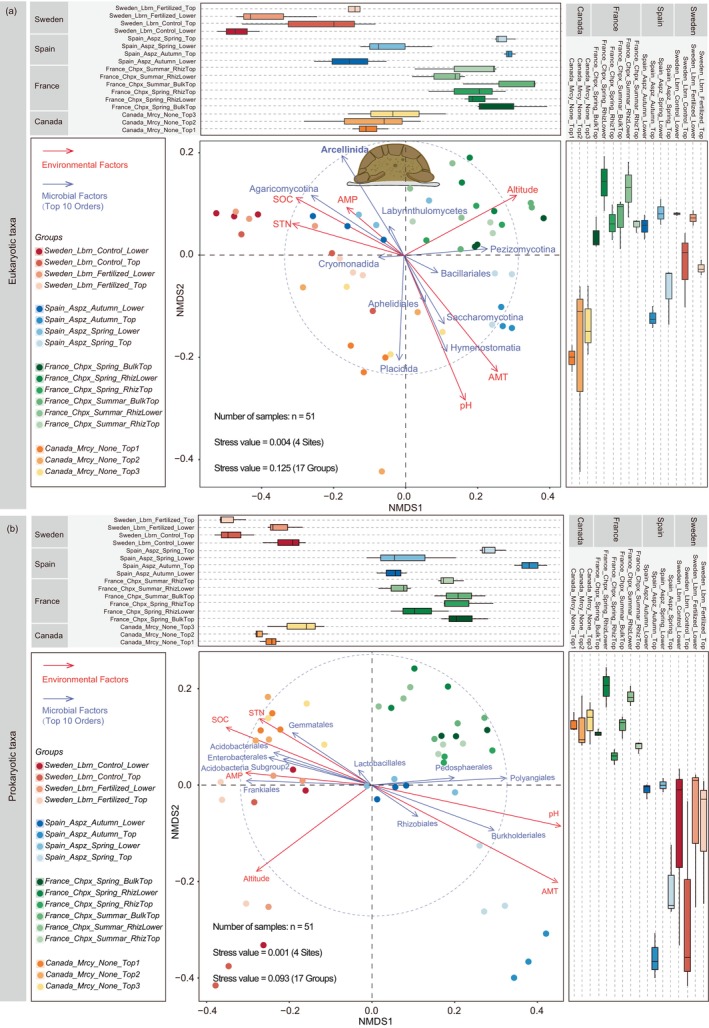
Non‐metric multidimensional scaling (NMDS) on the Bray‐Curtis distance between each sample. Dimensionality reduction of the Bray‐Curtis distance shows the eukaryotic (a) and prokaryotic (b) communities in forest soils. Dots in different colors (red, green, blue and yellow) represent sampling sites, while dots with varying transparency represent different treatments. Arrows show *envfit* results. Red and blue arrows represent the explanatory power of environmental factors and microbial groups (order level) on community composition, respectively.

To further assess the potential impact of soil environmental factors (altitude, pH, SOC, STN, AMP, and AMT) and microbial groups (the top 10 most abundant microbial orders) on microbial community composition in forest soil ecosystems, we performed non‐metric multidimensional scaling (NMDS) analyses for eukaryotic (Figure 3a) and prokaryotic (Figure 3b) rRNA sequence datasets based on Bray‐Curtis distances. For eukaryotic taxa, the coordinate points of microbial communities in different forest soils were significantly separated, with only minor overlap in sampling plots between France_Chpx and Spain_Aspz (Figure 3a). Moreover, soil pH (*R* ^2^ = 0.126, *p* < 0.001), AMT (*R* ^2^ = 0.116, *p* < 0.001), and SOC (*R* ^2^ = 0.041, *p* = 0.010) had significant effects on eukaryotic communities, explaining 12.6%, 11.6%, and 4.1% of the variation, respectively (Table S3). Arcellinida (*R* ^2^ = 0.066, *p* = 0.006) and Placidida (*R* ^2^ = 0.064, *p* = 0.009) accounted for 6.6% and 6.4% of the eukaryotic community composition, respectively. Hymenostomatia (*R* ^2^ = 0.057, *p* = 0.016) and Agaricomycotina (*R* ^2^ = 0.036, *p* = 0.048) had weaker structuring effects on the eukaryotic community composition, explaining 5.7% and 3.6% of the variation, respectively.

For prokaryotic taxa, the coordinate points representing microbial communities in various forest soils also showed significant separation, with minor overlap in sampling plots between the Canada_Mrcy and Sweden_Lbrn zones (Figure 3b). The prokaryotic diversity metric was highly correlated with AMT (*R* ^2^ = 0.092, *p* < 0.001), pH (*R* ^2^ = 0.077, *p* < 0.001), and altitude (*R* ^2^ = 0.042, *p* < 0.001), explaining 9.2%, 7.7%, and 4.2% of the variation, respectively (Table S3). Polyangiales (*R* ^2^ = 0.079, *p* = 0.001), Burkholderiales (*R* ^2^ = 0.062, *p* = 0.008), Frankiales (*R* ^2^ = 0.053, *p* = 0.011) and Acidobacteriales (*R* ^2^ = 0.030, *p* = 0.048) explained 7.9%, 6.2%, 5.3% and 4.8% of the prokaryotic community composition, respectively. All these taxa (eukaryotic and prokaryotic groups) were highly influenced by soil pH, explaining the variation in the communities of eukaryotes (12.6%) and prokaryotes (7.7%). In contrast, SOC, STN and AMP contributed to a lower extent, explaining marginal percentages (< 4.1%) of the variation in the three ordinations.

## 4 Discussion

Our findings showed that Arcellinida had the highest relative abundance (with an average of 12.6%) among eukaryotes at the order level (Figure 1). This result further confirms that Arcellinida, rather than individual fungal taxa, is the dominant eukaryotic order in forest ecosystems. Specifically, the relative abundance of Arcellinida was significantly higher at the Canadian site compared to other sites. We infer that this pattern is primarily driven by the local climate, characterized by a low mean annual temperature and high annual precipitation (Table S1), which promotes the accumulation of organic matter in forest soils. The influence of these climatic factors, particularly the mean annual temperature (*R* ^2^ = 0.116, *p* = 0.001), on the community diversity of eukaryotic taxa has also been demonstrated in our study (Figure 3a). Additionally, Arcellinida exhibited a preference for specific microhabitats within forest ecosystems, showing significantly higher relative abundance in the top soil layers compared to the lower layers. This suggests that Arcellinida spp. tend to aggregate in relatively stable environments, where the rich organic matter in the upper soil layers provides ample nutritional resources. Furthermore, this distribution pattern aligns with that of bacteria and fungi, reinforcing the idea that the distribution and abundance of predators are, to some extent, influenced by the availability of their prey. The findings from Berlinches de Gea et al. (2024) and Xiong et al. (2018) also confirm this pattern. Other studies have also highlighted that environmental and nutrient limitations not only restrict the distribution and growth rate of Arcellinida but also drive them to develop new survival strategies (Porfirio‐Sousa et al. 2024; Useros et al. 2023).

Moreover, prokaryotes (bacteria and archaea), which account for more than 70% of reads in our metatranscriptomic result (Table 1), are fundamental drivers of forest soil functioning. Major bacterial groups such as *Proteobacteria* (*Enterobacterales, Rhizobiales, Haliangiales*, etc.), *Acidobacteria* (*Acidobacteriales, Solibacterales*, etc.) *Actinobacteria* (*Frankiales, Solirubrobacterales*, etc.) and *Bacteroidota* (*Chitinophagales, Sphingobacteriales*, etc.) (Figure 1) play key roles in organic matter decomposition and the cycling of carbon, nitrogen and phosphorus, thereby regulating nutrient availability (Meng et al. 2024; Revillini et al. 2025) and energy flow (Fierer 2017; Goberna and Verdú 2018) in soil ecosystems.

In addition to their metabolic functions, prokaryotes structure microbial communities through both bottom‐up resource provision and top‐down regulation via predation by microbial eukaryotes (Groß et al. 2023). Such predation‐driven interactions can alter bacterial community composition and turnover, contributing to ecosystem stability and the maintenance of ecosystem functioning through functional redundancy and resilience (Tian et al. 2023). These processes provide an ecological context for our observation of abundant *Arcellinida* and highlight the importance of trophic interactions linking prokaryotic and eukaryotic communities.

In forest ecosystem systems, *Arcellinida* play important ecological and biogeochemical roles through complex interactions with bacteria and fungi, including symbiosis and diverse antagonisms, such as predation or competition (de Berlinches Gea et al. 2025). They regulate microbial community structure and influence nutrient cycling and ecosystem functioning (García‐Bodelón et al. 2025). Accordingly, their community composition and diversity show pronounced variation across microhabitats, soil types, and climatic gradients (Racoma et al. 2025).

In conclusion, we conclude that PCR‐ and primer‐based metabarcoding underestimates the abundance of a historically, morphologically well‐studied group. With metatranscriptomics this methodological hurdle does no longer exist and we encourage forest ecologists to use this method to achieve a more comprehensive overview of the microbial community in forest soils. In this study, we decided for a broad, higher‐taxonomy perspective, but in future species‐level analyses can be conducted.

## Author Contributions

**Longfei Kang:** writing – original draft, investigation, visualization, software, methodology, formal analysis, validation, funding acquisition. **Kenneth Dumack:** conceptualization, visualization, methodology, data curation, supervision, resources, project administration, funding acquisition, writing – review and editing.

## Conflicts of Interest

The authors declare no conflicts of interest.

## Supporting information


**Table S1:** Location and climatic description of the four coniferous forest sites.
**Table S2:** Soil types and bioSample description of the four coniferous forest sites.
**Table S3:** PERMANOVA results for taxonomic groups (eukaryotic and prokaryotic communities) across different environmental and biological factors.

**Table S4:** Order‐level taxonomic assignment of metatranscriptomic rRNA reads for prokaryotes and eukaryotes.

## Data Availability

The data that support the findings of this study are available in JGI Gold at https://gold.jgi.doe.gov/project?id=Gp0225829, reference number SAMN08776709–SAMN06265162. These data were derived from the following resources available in the public domain: Auer et al. (2024), https://doi.org/10.1111/nph.19471.
