# Supplementary material for: Protistan Predators Outshine Fungi in Forest Soil Activity
Source: J Eukaryot Microbiol. 2026 Mar 31;73(3):e70072. doi: 10.1111/jeu.70072 (PMC13037694; doi:10.1111/jeu.70072)
Supplement: Supplementary file 1 — Table S1: Location and climatic description of the four coniferous forest sites. Table S2: Soil types and bioSample description of the four coniferous forest sites. Table S3: PERMANOVA results for taxonomic groups (eukaryotic and prokaryotic communities) across different environmental and biological factors. [file JEU-73-e70072-s002.docx]

**Table S1. Location and climatic description of the four coniferous forest sites**

| **Site name** | **Spain_Aspz** | **France_Chpx** | **Canada_Mrcy** | **Sweden_Lbrn** |
| --- | --- | --- | --- | --- |
| Location | Aspurz | Champenoux | Montmorency | Lamborn |
| Country | Spain | France | Canada | Sweden |
| Coordinates | 42.70°N 1.14°W | 48.75°N 6.35°E | 47.27°N 71.21°W | 60.96°N 15.80°E |
| Collection year | 2012 | 2013 | 2016 | 2011 |
| Sample number | 12 | 18 | 9 | 12 |
| DTP | *Pinus sylvestris* | *Abies alba* | *Abies balsamea* | *Pinus sylvestris* |
| MAT | 12 | 13.7 | 0.4 | 7 |
| MAP | 913 | 778 | 1583 | 700 |
| Altitude | 615 | 254 | 777 | 273 |
| Climate | Submediterranean | Temperate | Boreal | Boreal |

Note: Site description including complete name, geographic origin, coordinates, year of collection, dominant tree species (DTP), mean annual temperature (MAT; °C) and mean annual precipitation (MAP; mm), altitude (meters) and climate.

**Table S2. Soil types and bioSample description of the four coniferous forest sites**

| **BioSample** | **Origin** | **Site name** | **Soil type** | **AMT** | **AMP** | **Altitude** | **SOC** | **STN** | **pH** |
| --- | --- | --- | --- | --- | --- | --- | --- | --- | --- |
| SAMN08776709 | Canada | Montmorency | organo-mineral soil | 0.4 | 1583 | 777 | 534.2 | 20.8 | 4.15 |
| SAMN08778090 | Canada | Montmorency | organo-mineral soil | 0.4 | 1583 | 777 | 493.3 | 16.4 | 3.82 |
| SAMN08778091 | Canada | Montmorency | organo-mineral soil | 0.4 | 1583 | 777 | 451.4 | 16.3 | 3.79 |
| SAMN09201744 | Canada | Montmorency | organo-mineral soil | 0.4 | 1583 | 777 | 482.8 | 21.9 | 4.1 |
| SAMN09200588 | Canada | Montmorency | organo-mineral soil | 0.4 | 1583 | 777 | 338.8 | 14.1 | 4.02 |
| SAMN09200587 | Canada | Montmorency | organo-mineral soil | 0.4 | 1583 | 777 | 405.4 | 16.5 | 3.96 |
| SAMN08777914 | Canada | Montmorency | organo-mineral soil | 0.4 | 1583 | 777 | 357.2 | 16.0 | 4.66 |
| SAMN08778092 | Canada | Montmorency | organo-mineral soil | 0.4 | 1583 | 777 | 428.3 | 18.8 | 4.03 |
| SAMN08778093 | Canada | Montmorency | organo-mineral soil | 0.4 | 1583 | 777 | 517.3 | 20.9 | 4.35 |
| SAMN07631444 | Sweden | Lamborn | organo-mineral soil | 7.0 | 700 | 273 | 70.42 | 2.35 | NA |
| SAMN07631445 | Sweden | Lamborn | organo-mineral soil | 7.0 | 700 | 273 | 70.42 | 2.35 | NA |
| SAMN07631446 | Sweden | Lamborn | organo-mineral soil | 7.0 | 700 | 273 | 70.42 | 2.35 | NA |
| SAMN07631447 | Sweden | Lamborn | organo-mineral soil | 7.0 | 700 | 273 | 101.7 | 2.35 | NA |
| SAMN07631448 | Sweden | Lamborn | organo-mineral soil | 7.0 | 700 | 273 | 101.7 | 2.35 | NA |
| SAMN07631449 | Sweden | Lamborn | organo-mineral soil | 7.0 | 700 | 273 | 101.7 | 2.35 | NA |
| SAMN07631468 | Sweden | Lamborn | mineral soil | 7.0 | 700 | 273 | 70.42 | 2.35 | NA |
| SAMN07631469 | Sweden | Lamborn | mineral soil | 7.0 | 700 | 273 | 70.42 | 2.35 | NA |
| SAMN07631470 | Sweden | Lamborn | mineral soil | 7.0 | 700 | 273 | 70.42 | 2.35 | NA |
| SAMN07631471 | Sweden | Lamborn | mineral soil | 7.0 | 700 | 273 | 101.74 | 2.35 | NA |
| SAMN07631472 | Sweden | Lamborn | mineral soil | 7.0 | 700 | 273 | 101.74 | 2.35 | NA |
| SAMN07631473 | Sweden | Lamborn | mineral soil | 7.0 | 700 | 273 | 101.74 | 2.35 | NA |
| SAMN07631311 | France | Champenoux | organo-mineral soil | 13.7 | 778 | 254 | 24.3 | 1.68 | 4.82 |
| SAMN07631314 | France | Champenoux | organo-mineral soil | 13.7 | 778 | 254 | 30.7 | 2.12 | 5.06 |
| SAMN07631309 | France | Champenoux | organo-mineral soil | 13.7 | 778 | 254 | 35.4 | 2.42 | 5.02 |
| SAMN07631308 | France | Champenoux | organo-mineral soil | 13.7 | 778 | 254 | 24.3 | 1.68 | 4.82 |
| SAMN07631297 | France | Champenoux | organo-mineral soil | 13.7 | 778 | 254 | 30.7 | 2.12 | 5.06 |
| SAMN07631310 | France | Champenoux | organo-mineral soil | 13.7 | 778 | 254 | 35.4 | 2.42 | 5.02 |
| SAMN07631288 | France | Champenoux | organic soil | 13.7 | 778 | 254 | 64.1 | 3.67 | 5.28 |
| SAMN07631292 | France | Champenoux | organic soil | 13.7 | 778 | 254 | 84.5 | 4.67 | 5.7 |
| SAMN07631301 | France | Champenoux | organic soil | 13.7 | 778 | 254 | 80.6 | 4.59 | 5.07 |
| SAMN07631298 | France | Champenoux | organic soil | 13.7 | 778 | 254 | 64.1 | 3.67 | 5.28 |
| SAMN07631318 | France | Champenoux | organic soil | 13.7 | 778 | 254 | 84.5 | 4.67 | 5.7 |
| SAMN07631291 | France | Champenoux | organic soil | 13.7 | 778 | 254 | 80.6 | 4.59 | 5.07 |
| SAMN07631320 | France | Champenoux | organic soil | 13.7 | 778 | 254 | 64.1 | 3.67 | 5.28 |
| SAMN06266375 | France | Champenoux | organic soil | 13.7 | 778 | 254 | 84.5 | 4.67 | 5.7 |
| SAMN07631290 | France | Champenoux | organic soil | 13.7 | 778 | 254 | 80.6 | 4.59 | 5.07 |
| SAMN07631315 | France | Champenoux | organic soil | 13.7 | 778 | 254 | 64.1 | 3.67 | 5.28 |
| SAMN07631317 | France | Champenoux | organic soil | 13.7 | 778 | 254 | 84.5 | 4.67 | 5.7 |
| SAMN07631313 | France | Champenoux | organic soil | 13.7 | 778 | 254 | 80.6 | 4.59 | 5.07 |
| SAMN06266137 | Spain | Aspurz | mineral soil | 12.0 | 913 | 615 | 46.7 | 1.70 | 5.12 |
| SAMN06266098 | Spain | Aspurz | mineral soil | 12.0 | 913 | 615 | 51.65 | 2.10 | 5.51 |
| SAMN06265045 | Spain | Aspurz | top soil (OL/OF/OH) | 12.0 | 913 | 615 | 70.05 | 2.55 | 4.72 |
| SAMN06264424 | Spain | Aspurz | top soil (OL/OF/OH) | 12.0 | 913 | 615 | 77.48 | 3.15 | 5.11 |
| SAMN06266095 | Spain | Aspurz | mineral soil | 12.0 | 913 | 615 | 55.8 | 1.60 | 4.37 |
| SAMN06266138 | Spain | Aspurz | mineral soil | 12.0 | 913 | 615 | 30.75 | 1.35 | 4.80 |
| SAMN06266097 | Spain | Aspurz | top soil (OL/OF/OH) | 12.0 | 913 | 615 | 83.7 | 2.40 | 3.97 |
| SAMN06266139 | Spain | Aspurz | top soil (OL/OF/OH) | 12.0 | 913 | 615 | 46.13 | 2.025 | 4.40 |
| SAMN06266096 | Spain | Aspurz | mineral soil | 12.0 | 913 | 615 | 35.5 | 1.80 | 5.36 |
| SAMN06266112 | Spain | Aspurz | mineral soil | 12.0 | 913 | 615 | 39.5 | 1.80 | 5.25 |
| SAMN06264762 | Spain | Aspurz | top soil (OL/OF/OH) | 12.0 | 913 | 615 | 53.25 | 2.70 | 4.96 |
| SAMN06265162 | Spain | Aspurz | top soil (OL/OF/OH) | 12.0 | 913 | 615 | 59.25 | 2.70 | 4.85 |

Note: MAT, mean annual temperature (°C); AMP, mean annual precipitation (mm); SOC, soil organic carbon; STN, soil total nitrogen.

**Table S3. PERMANOVA results for taxonomic groups (eukaryotic and prokaryotic communities) across different environmental and biological factors**

| **Groups** | **Factors** | **R^2^** | **Pr(>F)** | **Df** | **F** |
| --- | --- | --- | --- | --- | --- |
| Eukaryotes | AMT | 0.1160 | **0.0010** | 1 | 14.182 |
|  | AMP | 0.0117 | 0.2300 | 1 | 2.9166 |
|  | Altitude | 0.0360 | **0.0070** | 1 | 5.5394 |
|  | SOC | 0.0414 | **0.0100** | 1 | 6.1262 |
|  | STN | 0.0311 | 0.0610 | 1 | 5.0072 |
|  | pH | 0.1258 | **0.0010** | 1 | 15.238 |
|  | *Labyrinthulomycetes* | 0.0053 | 0.4160 | 1 | 1.8404 |
|  | *Bacillariales* | 0.0036 | 0.4630 | 1 | 1.7794 |
|  | *Cryomonadida* | 0.0011 | 0.5310 | 1 | 1.6896 |
|  | *Pezizomycotina* | 0.0126 | 0.1850 | 1 | 2.1020 |
|  | *Hymenostomatia* | 0.0566 | **0.0160** | 1 | 3.6889 |
|  | *Saccharomycotina* | 0.0298 | 0.0890 | 1 | 2.7220 |
|  | *Agaricomycotina* | 0.0366 | **0.0480** | 1 | 2.9682 |
|  | *Aphelidiales* | 0.0131 | 0.3940 | 1 | 2.1208 |
|  | *Placidida* | 0.0636 | **0.0090** | 1 | 3.9392 |
|  | *Arcellinida* | 0.0655 | **0.0060** | 1 | 4.0084 |
|  | Residuals | 0.3501 | / | / | / |
| Prokaryotes | AMT | 0.0920 | **0.0010** | 1 | 10.972 |
|  | AMP | 0.0202 | 0.0570 | 1 | 3.5829 |
|  | Altitude | 0.0420 | **0.0010** | 1 | 5.8292 |
|  | SOC | 0.0369 | **0.0040** | 1 | 5.2965 |
|  | STN | 0.0228 | **0.0270** | 1 | 3.8510 |
|  | pH | 0.0765 | **0.0010** | 1 | 9.3789 |
|  | *Lactobacillales* | 0.0011 | 0.4220 | 1 | 1.5393 |
|  | *Rhizobiales* | 0.0093 | 0.3180 | 1 | 1.8200 |
|  | *Gemmatales* | 0.0268 | 0.1200 | 1 | 2.4207 |
|  | *Polyangiales* | 0.0790 | **0.0010** | 1 | 4.2116 |
|  | *Acidobacteria_Subgroup_2* | 0.0318 | **0.0390** | 1 | 2.5911 |
|  | *Pedosphaerales* | 0.0194 | 0.2560 | 1 | 2.1660 |
|  | *Acidobacteriales* | 0.0301 | **0.0480** | 1 | 2.5321 |
|  | *Burkholderiales* | 0.0620 | **0.0080** | 1 | 3.6270 |
|  | *Enterobacterales* | 0.0238 | 0.1530 | 1 | 2.3178 |
|  | *Frankiales* | 0.0527 | **0.0110** | 1 | 3.3104 |
|  | Residuals | 0.3736 | / | / | / |

^1^ Values marked in bold indicate statistical significance (p < 0.05).

^2^ The number of sample is fifty-one for each predictor variable (n = 51).

^3^ Df, degrees of freedom R², R² values, F, F-statistics, Pr(>F), p-values.
